# HOW DO KOREANS PERCEIVE THE EXPERIENCE OF CAREGIVING? A SYSTEMATIC REVIEW OF THE OUTCOMES OF INFORMAL CAREGIVING

**DOI:** 10.1093/geroni/igae098.3854

**Published:** 2024-12-31

**Authors:** YoungBin Koh, Sunghyun Ko, Yeonjung Lee

**Affiliations:** Chung-Ang University, Seoul, Republic of Korea; Chung-Ang University, Seoul, Republic of Korea; Chung-Ang University, Seoul, Republic of Korea

## Abstract

Globally, several research have been conducted to show the evidence of caregiving impact. In general, caregiving responsibilities have negative impacts on caregivers’ well-being, and further research has investigated the differences in the outcomes in terms of gender, race, and socioeconomic status. However, there exists not enough efforts to explain these differences at a macro level. For example, how countries differ in the extent to which they rely on informal care services and whether there are differences in caregiving impact among countries. These differences might be related to differences in cultural and policy background. Specifically, South Korea promotes formal long-term care through its national insurance. However, the support for informal caregivers is somewhat weaker compared to other countries, and there is limited research. The purpose of this study is to evaluate the research evidence in Korean contexts and summarize current findings on the outcomes of informal caregiving. A systematic review was performed with a structured literature search following PRISMA guidelines in five Korean databases. Our systematic search yielded 1,143 articles, of which 31 were included for synthesis. Findings show evidence of a negative impact of caregiving including caregivers’ deteriorating physical and mental health as well as reduced employability, especially for female caregivers. However, there are also some positive aspects such as having more satisfied relationship with caregiver recipient. The findings underscore the need for interventions to reduce the negative impact, particularly among female caregivers. We also suggest further research to investigate the cultural contexts to make caregiving experience positive.

